# Somatostatin, Olfaction, and Neurodegeneration

**DOI:** 10.3389/fnins.2020.00096

**Published:** 2020-02-19

**Authors:** Daniel Saiz-Sanchez, Isabel Ubeda-Bañon, Alicia Flores-Cuadrado, Melania Gonzalez-Rodriguez, Sandra Villar-Conde, Veronica Astillero-Lopez, Alino Martinez-Marcos

**Affiliations:** Neuroplasticity and Neurodegeneration Laboratory, Ciudad Real Medical School, CRIB, University of Castilla-La Mancha, Ciudad Real, Spain

**Keywords:** α-synuclein, amyloid-β, anterior olfactory nucleus, hyposmia, tau

## Abstract

Alzheimer’s and Parkinson’s diseases are the most prevalent neurodegenerative disorders in aging. Hyposmia has been described as an early symptom that can precede cognitive and motor deficits by decades. Certain regions within the olfactory system, such as the anterior olfactory nucleus, display the neuropathological markers tau and amyloid-β or α-synuclein from the earliest stages of disease progression in a preferential manner. Specific neuronal subpopulations, namely those expressing somatostatin (SST), are preferentially affected throughout the olfactory and limbic systems. SST is a neuropeptide present in a subpopulation of GABAergic interneurons throughout the brain and its main function is to inhibit principal neurons and/or other interneurons. It has been reported that SST expression is reduced by 50% in Alzheimer’s disease and that it is related to the formation of Aβ oligomers. The mechanisms underlying the preferential vulnerability of SST-expressing neurons in Alzheimer’s disease (and, to a minor extent, in Parkinson’s disease) are not known but analysis of the available data could shed light on their etiology. This short review aims to update the knowledge of functional features of somatostatin within the olfactory system and its role in olfactory deficits during neurodegeneration.

## Somatostatin and Olfactory System

Somatostatin-14 (SST-14), a peptide composed of 14 amino acids, was first detected in the hypothalamus and was shown to inhibit the secretion of growth hormone (Brazeau et al., 1973). Later, SST-28 was identified in the intestine (Pradayrol et al., 1980). Both were derived from the same prohormone and showed similar affinity to at least six SST receptors (SSTR1–5, two isoforms SSTR2A–2B). These receptors are members of the G-protein-coupled seven-transmembrane domain receptor family, which are broadly expressed in the brain, including all olfactory structures (Olias et al., 2004).

Approximately 20% of neurons in the cerebral cortex are interneurons, most of them expressing γ-amino-butyric acid (GABA) and divided into three non-overlapping classes: parvalbumin-expressing, 5-HT3A receptor-expressing and somatostatin-expressing populations (Riedemann, 2019). This latter population can be, in turn, morphologically divided into Martinotti cells and non-Martinotti cells (among others, long-range projecting interneurons, basket cells and double-bouquet cells). Their axons typically target the distal dendrites of pyramidal cells (contrary to parvalbumin-expressing cells), creating a dense wiring into the local network with high basal firing activity that continues in the absence of synaptic input (Urban-Ciecko and Barth, 2016). The pivotal role of somatostatin-positive interneurons on disinhibition (inhibiting GABAergic interneurons and promoting pyramidal cell activation) has been recently demonstrated, and it appears to be essential for long-term regulation and network metaplasticity, which may be important for hippocampal-dependent learning and memory (Artinian and Lacaille, 2018). During development, cortical interneurons including somatostatin-positive cells derive from the medial ganglionic eminence (Hu et al., 2017), while those interneurons reaching the olfactory bulb originate in the lateral ganglionic eminence (Wichterle et al., 2001). Importantly, olfactory system together with the hippocampus constitutes a neurogenic niche during adulthood and specific interneuron population are periodically replaced (Lledo et al., 2008).

Olfactory sensory neurons are placed in the olfactory epithelium in the nasal cavity and send their axons to the olfactory bulb where primary olfactory information is processed. From here, the main cells (mitral and tufted cells) then send their projections to the rest of the olfactory cortices: anterior olfactory nucleus, olfactory tubercle, piriform cortex, amygdala, and entorhinal cortex (Martinez-Marcos, 2009). The anterior olfactory nucleus is a key structure that constitutes the first relay of olfactory information and it also sends ipsilateral and contralateral projections to the rest of olfactory areas (Brunjes et al., 2005). The piriform cortex is considered as the principal olfactory cortex and it is mainly involved in odor perception (Courtiol and Wilson, 2017). Specifically, the amygdala and the entorhinal cortices are multimodal areas that receive olfactory information, among others, and in turn send projections to the hippocampus (McDonald and Mott, 2017). This latter circuit participates in olfactory emotion and olfactory memory formation (Kadohisa, 2013).

**FIGURE 1:**
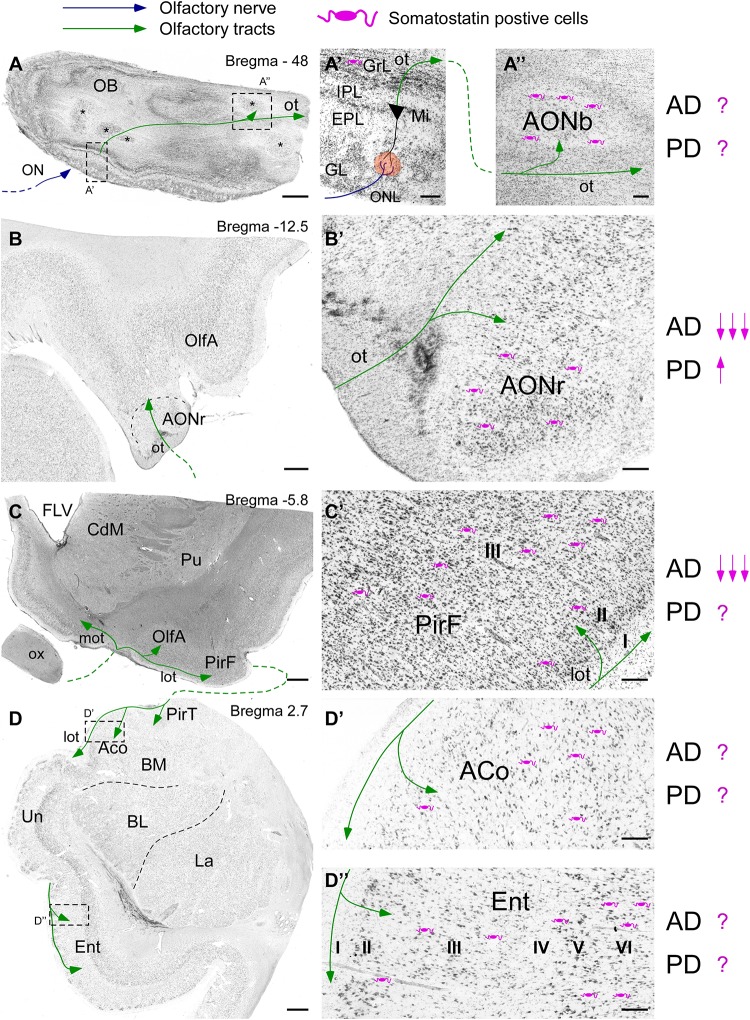
Scheme of the human olfactory system based on Nissl staining including the olfactory bulb **(A)** including different portions of the anterior olfactory nucleus (asterisks), the olfactory peduncle **(B)**, the piriform cortex **(C)**, the amygdala and the entorhinal cortex **(D)**. Bregma levels are indicated based on human brain atlas (Mai et al., 2015). Somatostatin cells (purple) localization and their variation regarding Alzheimer’s and Parkinson’s diseases are specified as described in the literature. High magnification images show **(A’)** the different layers and **(A”)** the anterior olfactory nucleus within olfactory bulb. Note the representation of the first olfactory relay placed in the glomeruli (orange circle) between the axons coming from the olfactory neurons forming the olfactory nerve (blue line) and the dendrites of mitral cells. Then, the mitral cells form the olfactory tract (green line), which projects over all olfactory areas. **(B’)** represents the site of entry of the references from mitral cells into cortex in the frontal lobe, where the retrobulbar portion of the anterior olfactory nucleus can be observed. Once the olfactory peduncle contacts with cortex two different olfactory tracts appear **(C)**; the medial olfactory tract (contralateral projections) and the lateral olfactory tract (ipsilateral projections). **(C’)** Represents the piriform cortex at its frontal subdivision. The typical three-layer histology is indicated. Note that somatostatin cells are in layer II and mainly in layer III. Afterward, olfactory tract reaches the temporal lobe **(D)**, including the temporal subdivision of the piriform cortex, the cortical amygdala **(D’)** and the most rostral portion of the entorhinal cortex **(D”)**. Note that no specific layer topography exists within the amygdala. On the contrary well defined six layers can be observed in the entorhinal cortex. Scale bars for **A,B** = 1000 μm; **C,D** = 2000 μm; and **A’–D”** = 200 μm.

Somatostatin -expressing cells are present in all olfactory areas as well as SSTRs. In mouse, SST is expressed in the granule cell layer and within the inner part of the external plexiform layer in the olfactory bulb (Lepousez et al., 2010a) and within the different subregions of the anterior olfactory nucleus (Brunjes et al., 2011). Regarding the olfactory cortices, SST-expressing cells are located mostly in layers II and III, while terminal axons target dendrites in layer I of the piriform cortex (Suzuki and Bekkers, 2010). In the amygdala, SST is present in deeper layers of olfactory cortical subregions, although non-olfactory central nucleus accumulates the highest levels (Real et al., 2009). The olfactory entorhinal cortex, thus corresponding to the lateral entorhinal cortex, contains large number of SST interneurons and fibers in all layers (Witter et al., 2017). On the other hand, SSTRs1–4 are specifically distributed within the olfactory bulb (Nocera et al., 2019) and they are highly expressed in the piriform cortex, olfactory amygdala, namely anterior cortical and posteromedial cortical nuclei and within the entorhinal cortex (Breder et al., 1992; Fehlmann et al., 2000; Hannon et al., 2002). Interestingly, SSTR5 is absent within the olfactory system with the exception of the olfactory tubercle (Feuerbach et al., 2000). In human brain, SST interneurons are present in all olfactory areas (Figure 1). In the olfactory bulb SST is sparse within the EPL while its expression is very abundant around the anterior olfactory nucleus (Figures 1A,A’,A”,B,B’; Smith et al., 1993). SST is also present in the piriform cortex, particularly in layers II and III (Saiz-Sanchez et al., 2015). Interestingly, the piriform cortex is divided into two different portions, namely the anterior portion placed at the frontal cortex and a posterior portion located at the temporal lobe (Figures 1C,C’). Temporal lobe also includes key olfactory areas involved in olfactory memory formation such as the amygdala and the entorhinal cortex (LaBar and Cabeza, 2006). SST is present in all amygdaloid nuclei including cortical olfactory amygdala (Unger et al., 1988) and in all layers of the entorhinal cortex (Figures 1D,D’,D”; Chan-Palay, 1987). SSTRs studies in human brain mostly show distribution patterns regarding neocortex and/or limbic system including the amygdala and the hippocampal formation, while olfactory system is still poorly examined (Reubi et al., 1986; Schindler et al., 1998).

Beside its cognitive and neuroendocrine functions (Viollet et al., 2008), central SST is involved in olfactory information processing such as olfactory detection and discrimination behaviors (Lepousez et al., 2010b; Nocera et al., 2019). Alternatively, SST contributes to additional actions related to olfaction, namely anxiety or fear-related behaviors (Yang et al., 2016). During the first relay of olfactory information SST regulates odor discrimination through SSTR2 receptor expressed by mitral cells (Lepousez et al., 2010b; Nocera et al., 2019). Furthermore, SST function related to the anterior olfactory nucleus, which constitutes the first relay from mitral cells, remains unknown. Piriform cortex is the main olfactory cortical area involved in the formation of odor percepts (Howard et al., 2009) and it regulates both excitation and inhibition network by inhibiting other interneurons, including other somatostatin cells and principal cells and they may help to discriminate odor responses from background cortical activity (Large et al., 2016). Moreover, SST cells regulate neurons by subtractive inhibition, which enhances the threshold for sensory input to trigger a response, that is independent of odor identity and intensity (Sturgill and Isaacson, 2015). Entorhinal cortex is involved in olfactory associative learning and recognition abilities (Nilssen et al., 2019). However, SST participation on olfactory processing within these areas is still poorly unknown. Nevertheless, SST distribution differs between mouse and rat (Brunjes et al., 2011).

## Somatostatin and Parkinson’s Disease

Olfactory deficits have been reported as a preclinical risk factor for the development of PD (Ponsen et al., 2004). Moreover, the main pathological marker (accumulation of intracellular aggregates of α-synuclein forming Lewy bodies) is present in key olfactory structures, such as the anterior olfactory nucleus, from the earliest stages of disease progression (Del Tredici and Braak, 2016).

Postmortem studies have reported that around 8% of cells containing Lewy pathology in the olfactory bulb are somatostatinergic cells, as compared to more than 50% of calcium-binding protein-expressing cells, particularly those expressing calbindin (CB) and calretinin (CR) and to a lesser extent parvalbumin-expressing cells (PV) (Ubeda-Banon et al., 2010). Studies comparing control vs. PD cases have revealed a significant decrease of SST and an increase of PV in the AON (Ubeda-Banon et al., 2017). These low percentages of co-localization of SST with Lewy pathology were also observed in the amygdala (Flores-Cuadrado et al., 2017). Studies carried out in control, non-demented and demented PD cases also suffering from Alzheimer’s disease demonstrated that only comorbid cases showed a 40% reduction of SST in the frontal and temporal cortices (Beal et al., 1986).

## Somatostatin and Alzheimer’s Disease

The two main hallmarks of AD are extracellular deposition of amyloid-β (Aβ_1__–__42_) forming senile plaques and intracellular aggregates of tau protein forming neurofibrillary tangles (Selkoe, 2001). Both have been described as affecting the olfactory system, while tau is involved early, especially in the entorhinal cortex (Braak et al., 2006) and within the anterior olfactory nucleus (Attems and Jellinger, 2006). These features make the olfactory system especially vulnerable to early stage disease progression.

Reduction of SST in AD was reported 40 years ago (Davies et al., 1980). Nowadays, SST reduction in the early stages and its involvement in memory formation is well established (Epelbaum et al., 2009). Other interneuron subpopulations such as CR or CB cells are reduced as well. Interestingly, as in PD, PV cells are not reduced in olfactory cortex (Saiz-Sanchez et al., 2015). However, most studies have focused on the hippocampal formation, and olfactory system studies are scarce. Moreover, olfactory deficits, namely hyposmia and anosmia, appear in patients with mild cognitive impairment (MCI) and correlate with later evolution to AD dementia (Devanand et al., 2000). Despite SST reduction being common within most olfactory areas (Saiz-Sanchez et al., 2010, 2015) olfactory explicit memory (namely odor identification and odor recognition, which are hippocampus dependent) is thought to be more deeply involved in AD as compared with olfactory threshold detection or implicit memory tasks such as habituation or sensitization (Quarmley et al., 2017). Nonetheless, the use of standardized tests remains controversial.

Somatostatin dysfunction is involved early in memory deficits observed in mouse models and may be affected by Aβ_1__–__42_ deposition (Schmid et al., 2016). Remarkably, SST is the main binder of Aβ_1__–__42_ and can encourage the formation of different Aβ_1__–__42_ oligomers by acquiring amyloid properties (Wang et al., 2017; Solarski et al., 2018). In agreement with this, SST and Aβ_1__–__42_ levels in the cerebrospinal fluid seem to be correlated (Duron et al., 2018) and co-localization of both in the human brain, including the olfactory cortex, are widespread histological features (Saiz-Sanchez et al., 2010, 2015). In fact, SST has been highlighted as a regulator of Aβ_1__–__42_ deposition (Saito et al., 2005). On the other hand, positron emission tomography results link olfactory impairment with tau rather than amyloid deposition (Risacher et al., 2017). However, we cannot rule out the aging influence on olfactory impairment and not only tau accumulation itself (Martel et al., 2015). Interestingly, cortistatin (a neuropeptide related to SST) can induce the phosphorylation of tau and may be linked with AD pathophysiology (Rubio et al., 2008).

## Overview

The olfactory system is early and severely affected by pathologic proteins in both AD and PD. On the other hand, SST is unequally involved, being strongly involved in AD and having a weaker effect in PD. SST is reduced during the early stages of AD, including the olfactory areas, and may be related to Aβ_1__–__42_ and/or tau pathophysiology. However, the knowledge of both SST and SSTRs involvement in the human olfactory system is very scarce. Olfactory deficits may be related to SST deficiencies and to memory impairment due to tau deposition. In fact, SST is preserved in non-demented PD cases. Finally, anterior olfactory nucleus highlights as a key olfactory area; it is one of the earliest affected by tau accumulation and contains high quantities of SST. Further studies may indicate which olfactory deficiencies are more accurate for early diagnoses and help to refine the scope of SST as a potential therapeutic target.

## Author Contributions

DS-S has coordinated all information, has written the manuscript, and has performed the figure. IU-B, AF-C, and SV-C have conducted the literature search focused on Parkinson’s disease and have written the related draft. MG-R and VA-L have conducted the literature search focused on Alzheimer’s disease and have written the related draft. AM-M has supervised all procedures.

## Conflict of Interest

The authors declare that the research was conducted in the absence of any commercial or financial relationships that could be construed as a potential conflict of interest.

## Abbreviations

ACo: cortical amygdala
AD: Alzheimer’s disease
AONb/AONr: anterior olfactory nucleus bulbar/retrobulbar portion
BL/BM: basolateral/basomedial amygdala
CdM: caudate nucleus medial
Ent: entorhinal cortex
EPL: external plexiform later
FLV: lateral ventricle frontal part
GL: glomerular layer
GrL: granule cell layer
IPL: internal plexiform layer
La: lateral amygdala
lot: lateral olfactory tract
Mi: mitral cell layer
mot: medial olfactory tract
OB: olfactory bulb
OlfA: olfactory area
ON: olfactory nerve
ot: olfactory tract
ox: optic chiasm
PirF/PirT: piriform cortex frontal/temporal subdivision
Pu: putamen
SST: somatostatin/somatostatin cells
Un: uncus.
